# Esophageal Transit, Contraction and Perception of Transit After Swallows of Two Viscous Boluses

**DOI:** 10.14740/gr687w

**Published:** 2015-10-21

**Authors:** Jucileia Dalmazo, Lilian Rose Otoboni Aprile, Roberto Oliveira Dantas

**Affiliations:** a University Center UNIVAG,Varzea Grande, MT, Brazil; b Medical School of Ribeirao Preto, USP, Ribeirao Preto, SP, Brazil

**Keywords:** Esophageal contraction, Esophageal transit, Bolus viscosity, Swallowing perception, Swallowing

## Abstract

**Background:**

There have been results showing the influence of bolus viscosities and consistency on esophageal motility and transit. However, there is no description about the influence of two different viscous boluses on esophageal contractions, bolus transit and perception of transit. Our objective in this investigation was to evaluate the esophageal transit and contraction after swallows of two viscous boluses.

**Methods:**

By impedance and manometric methods, we measured the esophageal transit and contraction after swallows of two viscous boluses of 5 mL volume, 100% barium sulfate and yogurt, swallowed in duplicate in the supine and upright positions. The bolus transit, esophageal contractions and the perception of bolus transit through the esophagus were evaluated in both positions. Impedance and contraction were measured at 5, 10, 15 and 20 cm from the lower esophageal sphincter. After each swallow, the volunteers were asked about the sensation of bolus transit through the esophagus.

**Results:**

In supine position, the yogurt had a less frequent complete bolus transit than barium. Also in the supine position, the esophageal transit was longer with yogurt than with barium. Esophageal contractions after swallows were similar between barium and yogurt boluses. There was no difference in perception of transit between the two boluses.

**Conclusion:**

Although both 100% barium sulfate and yogurt are viscous boluses and have similar viscosities, the transit through the esophagus is slower with yogurt bolus than with barium bolus, which suggests that viscosity may be not the sole factor to determine transit.

## Introduction

The characteristics of the swallowed boluses have influence on esophageal transit with bolus viscosity as one of them [1-5]. Compared with a non-viscous bolus, swallows of a viscous bolus cause a slowing in peristaltic propagation velocity and an increase in contraction duration [1], an increase in bolus transit duration [2], a decrease in contraction amplitude [2], a decrease in the complete bolus transit rate and, when measured by high-resolution manometry, does not cause alteration of integral distal contraction, distal esophageal amplitude and integrated relaxation pressure of the lower esophageal sphincter (LES) [3].

A viscous bolus is more likely to detect an esophageal motility abnormality than a liquid bolus [6, 7], indicating that a viscous bolus is more frequently associated with esophageal motility abnormalities. However, the viscosity of the bolus should not be the sole factor to determine alteration of esophageal motility and cause perception of the bolus transit through the esophagus. Perception of bolus transit is not always associated with alterations of esophageal transit or esophageal motility [4, 8-10]. Swallow of a viscous or solid bolus is more likely to be perceived than swallows of a liquid bolus [8, 10], but other characteristics of the swallowed bolus or the sensitivity of the subject may have influence on this perception.

Our aim in this investigation was to evaluate, in asymptomatic volunteers, the esophageal contractions, esophageal bolus transit, and perception of esophageal transit of a bolus of yogurt and a bolus of barium sulfate, both a viscous bolus, swallowed in the supine and in the sitting positions. Our hypothesis was that similar viscous boluses do not always cause similar esophageal contractions, transit duration and perception of transit.

## Materials and Methods

Esophageal contraction, transit and perception of transit were evaluated in 26 asymptomatic volunteers, 13 men and 13 women aged 18 - 60 years, mean 33.6 (12.2) years. They were the same volunteers in whom the effect of low viscosity and high viscosity boluses on esophageal motility and transit were previously evaluated [4]. They did not have digestive, pulmonary, neurologic disease, swallowing difficult, heartburn or regurgitation. They were recruited by advertisement inside the institution. The investigation was approved by the Human Research Committee of the University Hospital of Ribeirao Preto (SP, Brazil). All subjects gave written informed consent to participate in the investigation and the anonymity of each volunteer was preserved.

The methods for evaluation of esophageal motility and transit were the same as previously described [4]. Esophageal contraction and transit were measured with a manometric and impedance catheter, Sandhill Scientific Manometry System (Highlands Ranch, CO, USA) that has five pressure and four impedance-measuring segments [11], with pressure transducers at a distance of 5 cm apart, and metal rings for measurement of impedance placed 2 cm apart, centered at the pressure transducers. The amplitude, duration and area under the curve (AUC) of the contractions, and the time of propagation of peristaltic contractions from 20 to 5 cm from the LES were analyzed on the manometric tracings. Ineffective esophageal contractions occur when there was no peristaltic contraction after swallow or the peristaltic contraction was of amplitude below 35 mm Hg. The total bolus transit time (TBTT), bolus head advance time (BHAT), and segment transit time (STT) were analyzed on the impedance tracings, as previously described [11].

The volunteers were studied in the sequence of sitting and supine positions. The catheter was introduced through the nose and was anchored when the distal pressure sensor registered the LES pressure. The other pressure and impedance sensors registered the values at 5, 10, 15, and 20 cm from the LES. After 5 min of stabilization of the recording, each volunteer swallowed twice in a random bolus sequence, in the sitting and supine positions, 5 mL of yogurt (Integral Natural Yogurt Nestle, Sao Paulo, SP, Brazil), a colloidal system food, and 5 mL of barium sulfate (Bariogel 100% Laboratory Cristalia, Itapira, SP, Brazil), used for radiological digestive examination, a suspension with 1 g of barium sulfate for each 1 mL of water, both at room temperature (25 - 30 °C). After the completion of each swallow, subjects were asked about their perception of the bolus passage using a scoring system [8]: 1 - bolus passage without perception of transit; 2 - slow transit; 3 - partial blockage; 4 - complete blockage. The subjects performed 104 swallows of yogurt and 104 swallows of barium sulfate, 52 swallows in each position for each bolus.

The yogurt had a pH of 4.6 and a density of 1.01 g/cm^3^, and the barium sulfate had a pH of 7.9 and a density of 0.68 g/cm^3^. The viscosity of the yogurt and barium sulfate boluses was measured with a rheometer (Brookfield Engineering Laboratories, MA, USA) with the spindle LV-3.

Statistical analysis was done by the Center of Quantitative Analysis of the Medical School of Ribeirao Preto USP (CEMEQ) using a linear model with mixed effects [12]. The model was adjusted using the Proc Mixed feature of the SAS software package version. The McNemar test and X^2^ test were also used. The results are reported as mean and standard deviation (SD), unless otherwise stated. The differences were considered significant when P ≤ 0.05 in a two-tailed statistical analysis.

## Results

**Figure 1:**
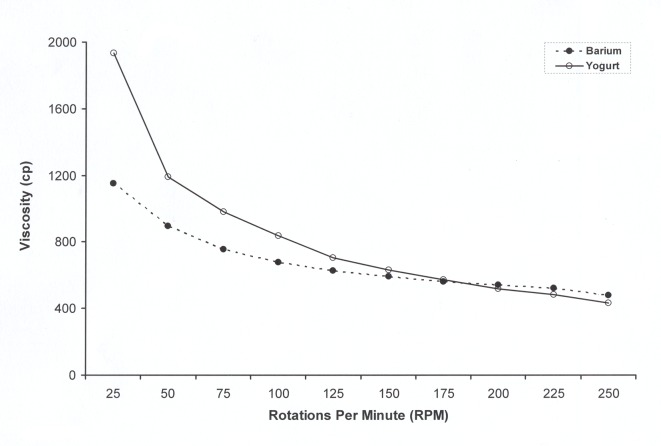
Measurement of viscosity, in centipoise (cp), of the barium and the yogurt, with the spindle rotation from 25 to 250 rotations per minute.

The viscosity measurement at 26 °C with spindle rotation from 25 to 250 rpm found a range of values from 1,934 centipoise (cp) to 432 cp for the yogurt and a range of values from 1,152 to 480 cp for the barium sulfate (Fig. 1).

The impedance sensors of the catheter registered, in the sitting position, a complete bolus transit in 48% of swallows of yogurt and 48% of swallows of barium boluses (P > 0.05) and, in the supine position, a complete bolus transit in 52% of swallows of yogurt and 77% of swallows of barium (P < 0.05). The TBTT, in sitting position, was similar with the yogurt (7.9 (2.0) s) and barium boluses (7.7 (1.8) s) (P > 0.05), but in the supine position, the transit was longer with yogurt (9.9 (2.2) s) than with barium (8.6 (2.2) s) (P < 0.01). The transit was longer in the supine position than in sitting position for barium and yogurt boluses (P < 0.02).

BHAT was longer with the yogurt bolus than with the barium bolus with swallows performed in the sitting position (P < 0.04), and in the proximal esophageal body in the supine position (P < 0.04, Table 1). For the STT, it was longer for yogurt bolus in the proximal esophagus in the supine position (P < 0.03, Table 1).

**Table 1:** Bolus Head Advanced Time (BHAT) and Segment Transit Time (STT), in Seconds, in the Sitting and Supine Positions After Swallows of Yogurt and Barium Sulfate, Measured From 20 - 15 cm, 15 - 10 cm, 10 - 5 cm From the Lower Esophageal Sphincter

	Sitting, mean (SD)			Supine, mean (SD)		
	20 - 15 cm	15 - 10 cm	10 - 5 cm	20 - 15 cm	15 - 10 cm	10 - 5 cm
BHAT						
Yogurt	0.9 (1.4)*	2.0 (2.0)*	2.1 (1.5)*	1.0 (0.7)*	1.9 (1.2)	3.0 (2.7)
Barium	0.5 (0.5)	1.1 (1.2)	1.2 (0.7)	0.8 (1.2)	1.4 (0.6)	2.5 (1.4)
STT						
Yogurt	5.9 (3.2)	5.2 (2.8)	5.2 (1.7)	6.0 (2.8)*	6.1 (2.7)	6.6 (2.4)
Barium	5.1 (2.7)	5.4 (2.8)	5.7 (2.9)	4.8 (2.5)	5.9 (1.6)	6.2 (1.0)

*P < 0.04 vs. barium.

Esophageal contractions were similar for yogurt bolus and barium bolus (Table 2), except in the distal esophagus with the subjects supine, where the amplitude of contractions was higher with the barium bolus compared with yogurt bolus (P < 0.05). There was no difference between yogurt and barium boluses in the duration of propagation of esophageal peristaltic contraction (sitting: yogurt - 3.9 (2.9) s, barium - 3.2 (1.6) s; supine: yogurt - 3.6 (4.0) s, barium - 2.7 (2.6) s, P > 0.05). The results for amplitude and AUC were higher in the supine position than in sitting position (P < 0.02, Table 2). There were no simultaneous contractions after yogurt and barium swallows.

**Table 2:** Amplitude (mm Hg), Duration (seconds) and Area Under the Curve (AUC, mm Hg × s) of Esophageal Contractions After Swallows of Yogurt and Barium Sulfate, Measured at 20, 15, 10 and 5 cm From the Lower Esophageal Sphincter in the Sitting and Supine Positions

	Sitting, mean (SD)		Supine, mean (SD)	
	Yogurt	Barium	Yogurt	Barium
Amplitude				
20 cm	70.1 (40.1)	64.9 (36.0)	81.8 (37.9)	87.6 (37.9)
15 cm	43.7 (29.3)	41.0 (28.3)	60.9 (41.4)	65.0 (42.3)
10 cm	73.6 (44.7)	69.3 (42.5)	95.2 (60.1)	101.7(53.1)
5 cm	107.8 (55.4)	98.3 (57.6)	110.2 (63.2)*	130.7 (69.4)
Duration				
20 cm	2.4 (1.0)	2.3 (0.8)	2.8 (1.3)	2.8 (1.2)
15 cm	2.4 (0.8)	2.4 (1.0)	2.9 (1.1)	2.8 (0.9)
10 cm	2.9 (1.2)	2.7 (1.0)	3.0 (1.3)	3.3 (1.3)
5 cm	3.1 (1.4)	3.3 (1.5)	3.5 (1.3)	3.7 (1.6)
AUC				
20 cm	100.6 (67.6)	87.7 (52.1)	150.8 (107.0)	149.8 (91.5)
15 cm	69.2 (52.8)	70.4 (57.6)	112.1 (80.9)	118.9 (87.0)
10 cm	130.2 (85.6)	118.8 (89.0)	182.4 (145.1)	202.6 (149.1)
5 cm	205.6 (173.1)	216.2 (210.0)	237.0 (189.6)	294.0 (267.0)

*P < 0.01 vs. barium.

The number of ineffective esophageal contractions was higher with swallows of yogurt than with swallows of barium (Table 3), almost significant in the sitting position (yogurt: 36.5%, barium: 17.3%, P = 0.06) and significant in the supine position (yogurt: 21.2%, barium: 9.6%, P = 0.03).

**Table 3:** Number of Ineffective Esophageal Contractions After Swallows of Yogurt and Swallows of Barium Performed in the Sitting and Supine Positions

	Sitting				Supine			
	Yes		No		Yes		No	
	N	%	N	%	N	%	N	%
Yogurt	19*	36.5	33	63.5	11**	21.2	41	78.8
Barium	9	17.3	43	82.7	5	9.6	47	90.4

*P = 0.06 vs. barium. **P = 0.03 vs. barium.

The frequency of perception of bolus transit (grades 2 and 3 of the scoring system) was similar between yogurt and barium in the sitting position (yogurt: 39.6%, barium: 23.1%, P = 0.32) and supine position (yogurt: 28.8%, barium: 14.3%, P = 0.06), although in this position, the difference was almost significant (Table 4). None swallow was followed by grade 4 of the scoring system.

**Table 4:** Perception of Bolus Transit After Swallows of Yogurt and Barium Boluses in the Sitting and Supine Positions

	Sitting				Supine			
	Yes		No		Yes		No	
	N	%	N	%	N	%	N	%
Yogurt	18	39.6	34	65.4	15*	28.8	37	71.2
Barium	12	23.1	40	76.9	9	14.3	43	82.7

*P = 0.06 vs. barium.

There was no association between the occurrence of ineffective esophageal contractions and the perception of bolus transit, with yogurt (sitting: P = 0.12, supine: P = 0.56) and barium (sitting: P = 0.84, supine: P = 0.44).

## Discussion

There was difference between yogurt and barium in the transit through the esophagus. The yogurt bolus, which has a higher density and viscosity in situation of slower transit, has a longer esophageal transit and a more frequent incomplete bolus transit. The esophageal contractions did not have difference between the bolus, except in distal esophagus in supine position. The position of the subjects has influence on esophageal contractions and transit. In the supine position, the transit was longer, and the contraction amplitude and AUC of contractions were higher than in the sitting position.

The results suggested that there is a slower esophageal transit with yogurt than with barium, without differences in contraction amplitude. This more difficult transit is not associated with an increase in bolus perception frequency. In the sitting position, it is possible that the perception is more frequent with yogurt, but the difference did not reach significance.

In upright position, a viscous bolus has a longer pharyngeal transit duration than a non-viscous bolus [13-15]. In esophagus, a longer transit was described with the viscous bolus in the supine position [3], in the upright position [4] and in both positions [2]. In the comparison between viscous and non-viscous bolus, the higher viscosity of the bolus should cause a higher friction force in the pharynx and esophagus and cause a slower transit compared with a non-viscous bolus. However, yogurt and barium sulfate are viscous bolus with viscosity difference only during slow transit, which may be enough to cause that the BHAT to be slower with yogurt than with barium. This slower bolus head transit may be consequence of the transit through the pharynx, by the action of the “pharyngeal pump” which is important for esophageal transit [16]. The esophageal STT was not different when comparing yogurt with barium.

Ineffective esophageal contractions could be an explanation for differences in esophageal transit, which has a higher frequency of occurrence after yogurt swallows than barium swallows. However, other characteristics of the bolus may also be the cause for some observed differences in bolus transit.

Gravitational force should have the same action in the yogurt and barium, so in the upright position, the differences between the bolus could be a consequence of the bolus characteristics. In the supine position, the transit is slower than in upright position, which is associated with an increase in contraction amplitude seen with both boluses, which should be an alteration to compensate the loss of gravitational force.

The number of ineffective esophageal contractions was not associated with the perception of bolus transit, but perception may be consequence of the longer bolus transit. The factors that are associated with the perception of bolus transit through esophagus are esophageal contraction impairment, longer bolus transit, increase in intrabolus pressure [5] and the sensitivity of the individuals [9]. The increase in bolus pressurization is explained by the characteristic of the bolus, the increase in esophageal smooth muscle length tension and higher esophageal contraction pressure [5]. However, a relationship between motility, transit and perception of transit was not found in patients with gastroesophageal reflux disease and in subjects whose contraction was changed from effective to ineffective by the use of sildenafil, a drug that increases the intracellular cyclic guanosine monophosphate and cause a reversible reduction in the amplitude of peristaltic esophageal contractions [9].

Swallows of a more consistent bolus, as a solid bolus, increase the contraction amplitude [8], a situation that should improve the esophageal transit and avoid transit perception. As the boluses examined were viscous bolus, differences between them in terms of contraction and transit were not expected.

Experimental conditions of rheological measurements may introduce errors on results, which may be different than expected [17]. Viscosity and viscoelasticity rheological properties depend upon the external condition applied, such as stress, strain, timescale and temperature, and the internal sample variation [18, 19]. With these situations, it is difficult to establish a particular food characteristic for treatment of a patient. The higher density of the bolus also increases the transit duration, at least in the pharynx [20, 21].

In conclusion, the two boluses evaluated, barium sulfate and yogurt, both viscous boluses, had different bolus transit, suggesting that not only viscosity has influence on transit.
